# Tissue Engineered Axon Tracts Serve as Living Scaffolds to Accelerate Axonal Regeneration and Functional Recovery Following Peripheral Nerve Injury in Rats

**DOI:** 10.3389/fbioe.2020.00492

**Published:** 2020-05-25

**Authors:** Kritika S. Katiyar, Laura A. Struzyna, Joseph P. Morand, Justin C. Burrell, Basak Clements, Franco A. Laimo, Kevin D. Browne, Joachim Kohn, Zarina Ali, Harry C. Ledebur, Douglas H. Smith, D. Kacy Cullen

**Affiliations:** ^1^Center for Brain Injury and Repair, Department of Neurosurgery, Perelman School of Medicine, University of Pennsylvania, Philadelphia, PA, United States; ^2^Center for Neurotrauma, Neurodegeneration & Restoration, Corporal Michael J. Crescenz Veterans Affairs Medical Center, Philadelphia, PA, United States; ^3^Axonova Medical LLC, Philadelphia, PA, United States; ^4^Department of Bioengineering, School of Engineering and Applied Science, University of Pennsylvania, Philadelphia, PA, United States; ^5^New Jersey Center for Biomaterials, Rutgers, The State University of New Jersey, Piscataway, NJ, United States

**Keywords:** neural tissue engineering, axon stretch growth, axon guidance, axon regeneration, tissue engineered nerve graft

## Abstract

Strategies to accelerate the rate of axon regeneration would improve functional recovery following peripheral nerve injury, in particular for cases involving segmental nerve defects. We are advancing tissue engineered nerve grafts (TENGs) comprised of long, aligned, centimeter-scale axon tracts developed by the controlled process of axon “stretch-growth” in custom mechanobioreactors. The current study used a rat sciatic nerve model to investigate the mechanisms of axon regeneration across nerve gaps bridged by TENGs as well as the extent of functional recovery compared to nerve guidance tubes (NGT) or autografts. We established that host axon growth occurred directly along TENG axons, which mimicked the action of “pioneer” axons during development by providing directed cues for accelerated outgrowth. Indeed, axon regeneration rates across TENGs were 3–4 fold faster than NGTs and equivalent to autografts. The infiltration of host Schwann cells – traditional drivers of peripheral axon regeneration – was also accelerated and progressed directly along TENG axons. Moreover, TENG repairs resulted in functional recovery levels equivalent to autografts, with both several-fold superior to NGTs. These findings demonstrate that engineered axon tracts serve as “living scaffolds” to guide host axon outgrowth by a new mechanism – which we term “axon-facilitated axon regeneration” – that leads to enhanced functional recovery.

## Introduction

Peripheral nerve injuries (PNIs) present a serious medical concern, with over 550,000 neurosurgical procedures in the United States and Europe annually (Robinson, 2000; Evans, 2001; Siemionow and Brzezicki, 2009; Brattain, 2012). PNI is also a major health concern for combat personnel as extremity trauma accounts for as much as 79% of trauma cases in wounded warriors treated in United States military facilities, and combat-related blast and/or penetrating injuries generally result in major tissue and peripheral nerve loss (Owens et al., 2008; Lew et al., 2010; White et al., 2011; Stannard et al., 2012). Despite the large number of afflicted patients, only 50% achieve good to normal restoration of function following surgical repair – regardless of the repair strategy (Scholz et al., 2009). This is partly due to insufficient PNI surgical repair strategies that lack biologically active guidance cues necessary to drive long distance regeneration. Thus, there is a clear need for pro-regenerative “bridges” across segmental nerve defects capable of accelerating axonal regeneration – a major rate-limiting step to the extent of functional recovery.

Following complete nerve transection the axonal segments distal to the injury site rapidly degenerate within hours to days. This is followed by a gradual loss of supportive cells (e.g., Schwann cells) that otherwise serve as a natural labeled pathway necessary to guide axon outgrowth to end targets. For long distance axon regeneration, such as down a nerve in the arm, there is a race against time as the slow growth of regenerating axons (approximately 1 mm/day) is outpaced by the gradual disappearance of the physical and chemical guidance cues necessary to guide that regeneration (Lee and Wolfe, 2000; Hall, 2001; Belkas et al., 2004). This commonly results in poor recovery of motor function distal to the original nerve injury (Evans, 2000; Meek and Coert, 2002).

Despite significant efforts, PNI repair strategies have not progressed beyond nerve guidance tubes (NGTs) or acellular nerve allografts (ANAs) to bridge small gaps, and autografts for larger defects – with each strategy having notable shortcomings (Pfister et al., 2011). For instance, the use of autografts involves harvesting healthy, otherwise uninjured nerve to serve as a living bridge for host axon regeneration, and results in donor site morbidity as well as other complications (Pfister et al., 2011). Allografts are a promising alternative, and while providing living support cells and structure for re-growth and re-vascularization, they require immunosuppression and have limited availability (Mazzocca and Lindsay, 2018). While NGTs and ANAs are available as “off-the-shelf,” these result in limited functional regeneration and therefore are generally only used for short-gap repairs of non-critical sensory nerves close to the end target (Mazzocca and Lindsay, 2018). As a result, the field is in need of an alternative technology to promote rapid axon regeneration across segmental defects while providing a mechanism to attenuate the loss of support cells in the distal nerve segment following major PNI.

**FIGURE 1:**
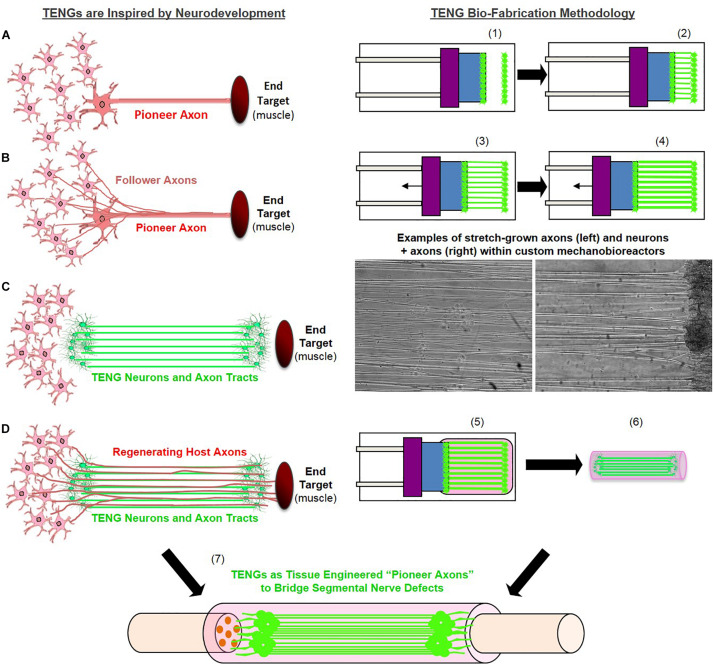
Tissue Engineered Nerve Graft (TENG) Inspiration, Biofabrication, and Surgical Implementation. *LEFT:* TENGs are inspired by axonal pathfinding during nervous system development, where **(A)** *“pioneer axons”* reach a target first, and then **(B)** serve as a physical guide for “follower axons” to reach that target. TENG axons are effectively **(C)** *tissue engineered “pioneer axons,”* thereby functioning as a **(D)** *living scaffold* to direct and target regenerating host axons across segmental nerve defects. *RIGHT:* TENGs are biofabricated in custom mechanobioreactors via the process of axon “stretch-growth.” Fully formed TENGs – comprised of longitudinally aligned axons encased in a collagenous matrix and rolled into a tubular form – are used to physically bridge segmental defects in peripheral nerve. Briefly, (1) Primary DRG neurons are plated in custom mechanobioreactors. (2) Traditional axon outgrowth integrates two neuron populations. (3) A computer-controlled micro-stepper motor is engaged to gradually separate the two neuron populations, applying mechanical tension to spanning axons. (4) Tension induces axon “stretch-growth,” resulting in increased length, diameter, and fasciculation. “Stretch-growth” occurs for days to weeks at 1–10 mm/day, depending on desired length. (5) Immediately prior to implant, neurons and stretch-grown axons are encased in ECM for stabilization. (6) The ECM containing neurons and stretch-grown axons is “rolled” and transferred into an NGT. (7) NGT containing the cylindrical TENG (neurons/axons embedded in ECM) is then sutured to sciatic nerve to bridge an excised segment.

We have developed living tissue engineered nerve grafts (TENGs), which are lab-grown nervous tissue comprised of long, aligned axonal tracts spanning two populations of dorsal root ganglia (DRG) neurons. The ability to generate TENGs is based upon the process of axon growth via continuous mechanical tension or “stretch growth” (Smith et al., 2001). Stretch growth is a natural axon growth mechanism that can extend axons at rapid rates without the aid of chemical cues, physical guides or growth cones. We routinely replicate this process in custom-built mechanobioreactors through the controlled separation of two integrated neuronal populations (Figure 1). During stretch growth, individual axons gradually coalesce with neighboring axons to form large axon tracts, or fascicles, taking on a highly organized parallel orientation. TENGs are subsequently created by embedding these living axon tracts in a three-dimensional (3D) matrix and removing them *en masse* for transplantation (Pfister et al., 2006; Huang et al., 2009). This unique platform can generate axons of unprecedented lengths in a very short time frame (5–10 cm in 14–21 days, with no theoretical limit as to the final axon length) from a range of neuronal subtypes and species (Smith et al., 2001; Pfister et al., 2004; Huang et al., 2008; Smith, 2009).

We have previously transplanted TENGs to study regeneration in a rodent PNI model (Huang et al., 2009), as well as in a rodent spinal cord injury model (Iwata et al., 2006), with each study demonstrating TENG survival over weeks to months absent any immune suppressive regime. Although these results were promising, for the particular case of PNI repair we did not uncover the mechanism(s) by which TENGs affected axon regeneration, nor did we measure the performance of TENGs compared to the two clinical standards for PNI repair: NGTs and autografts. Therefore, the objective of this study was to investigate the mechanism-of-action (MoA) by which TENGs facilitate host axonal regeneration and Schwann cell (SC) infiltration as well as to determine the efficacy of TENGs as compared to standard clinical techniques.

The inspiration for the regenerative MoA of TENGs was based on the observation of axon growth directly along so-called “pioneer” axons during nervous system development. In this case, first, pioneer axons employ pathfinding strategies to find the optimal course to reach and synapse with appropriate targets. Presumably, changes occur on the shaft of the pioneer axons that provide structural cues to direct targeted axon outgrowth from other neurons in the originating site (Figure 1). Thus, we hypothesized that like pioneer axons, TENGs would provide cues to promote host regeneration by direct host:TENG axon–axon interactions, ultimately accelerating host axon regeneration across segmental nerve defects and facilitating target reinnervation. We also hypothesized that TENG axons would grow out distally to penetrate into the host nerve, thereby extending this living labeled pathway for regeneration. In the current study, we found that TENGs served as a living scaffold to promote functional restoration at levels surpassing those of NGTs alone and at least equivalent to reverse autografts. Ultimately, tissue engineered “living scaffolds” exploiting potent developmentally-inspired mechanisms of regeneration may be useful to facilitate functional recovery following neurotrauma or neurodegenerative disease.

## Materials and Methods

All procedures were approved by the Institutional Animal Care and Use Committees at the University of Pennsylvania and the Michael J. Crescenz Veterans Affairs Medical Center and adhered to the guidelines set forth in the NIH Public Health Service Policy on Humane Care and Use of Laboratory Animals (2015).

### Biofabrication of Tissue Engineered Nerve Grafts

TENGs were generated using dorsal root ganglia (DRG) neurons isolated from embryonic day 16 fetuses from timed-pregnant Sprague-Dawley dams (Charles River). Whole DRG explants were cultured in Neurobasal^©^ medium supplemented with 2% B-27, 500 μM L-glutamine, 1% FBS (Atlanta Biologicals), 2.5 mg/mL glucose (Sigma), 20 ng/mL 2.5S nerve growth factor (BD Biosciences), 20 mM 5FdU (Sigma), and 20 mM uridine (Sigma). Cultures were transduced to express mCherry (AAV1-CB7-CI-mCherry.WPRE.rBG, UPenn Vector Core) or GFP with an AAV viral vector (AAV2/1.hSynapsin.EGFP.WPRE.bGH, UPenn Vector Core).

Explants were plated into mechanical elongation chambers custom-fabricated for stretch-growth. These chambers contained two adjoining membranes of 33C Aclar (SPI supplies) treated with 20 μg/mL Poly-D-lysine (BD Biosciences) and 20 μg/mL laminin (BD Biosciences). One of these membranes, denoted the “towing membrane,” could be precisely moved by a stepper motor. The DRGs were plated in two populations on either side of the membrane interface to allow formation of axonal networks between these two populations. The stepper motor system then separated the populations in micron-size increments until the TENGs reached their desired lengths. Stretched cultures were encapsulated in an extracellular matrix (ECM) comprised of 3.0 mg/mL rat-tail collagen type I (BD Biosciences) supplemented with 1.0 ug/mL 2.5S nerve growth factor (BD Biosciences). After gelation at 37°C, embedded cultures were manipulated into cylinders, removed from the membranes, and placed within either a premeasured NeuroFlex^TM^ (collagen; Stryker), NeuroTube^TM^ (polyglycolic acid or PGA; Baxter/Synovis), or tyrosine-derived polycarbonate (TyrPC; Rutgers University) NGT (Magno et al., 2010). TyrPC NGTs were synthesized from braided TyrPC fibers (80–100 μm diameter) and dip-coated with a hyaluronan solution (HyStem) (Ezra et al., 2013; Clements et al., 2016).

### Peripheral Nerve Surgery and Repair

Experimental subjects were adult male rats (Sprague-Dawley, Charles River). Rats were anesthetized using 2.5% inhaled isoflurane. The left rat sciatic nerve was exposed and a 1.0 or 2.0 cm segment was excised and replaced with an autologous nerve graft (1.0 cm long only; reversal of excised nerve), an NGT (1.2 cm long only; filled with the same ECM + NGF used to encapsulate TENGs), or a TENG within an NGT (1.2 cm or 2.2 cm long). There was no difference in the acute regenerative performance of TENGs encased with the PGA (NeuroTube), collagen (NeuroFlex), or TyrPC NGTs, so these groups were combined for statistical purposes. All chronic TENGs were encased in collagen (NeuroFlex) NGTs. Constructs were implanted by inserting the two nerve stumps into the NGT’s ends (1 mm overlap, leaving a gap length of 1.0 or 2.0 cm long), which were sutured to the epineurium using four 8–0 absorbable sutures. The wound site was closed with 4–0 prolene or nylon sutures. Experimental groups and group sizes were as follows for sciatic nerve lesion in wild-type rats (unless otherwise indicated), repaired using Autograft (1.0 cm gap: *n* = 8 at 2 weeks, *n* = 4 at 16 weeks), NGT (1.0 cm gap: *n* = 6 at 2 weeks, *n* = 3 at 16 weeks), TENGs (1.0 cm gap: *n* = 6 at 2 weeks into wild-type host, *n* = 3 at 2 weeks into GFP + host, *n* = 4 at 16 weeks; 2.0 cm gap: *n* = 4 at 12 weeks, *n* = 5 at 16 weeks).

### Functional Assessment

Electrophysiological response was measured at 12 and/or 16 weeks post-transplantation to determine functional regeneration of the nerve following repair. At the terminal time point, animals were re-anesthetized, the surgical site was re-exposed to measure compound nerve action potential (CNAP) and compound muscle action potential (CMAP) recordings.

Compound muscle action potential recordings were obtained from the tibialis anterior with a bipolar subdermal recording electrode, and a ground electrode (Medtronic, Jacksonville, FL; #8227103) was inserted into the tendon. The nerve was stimulated (biphasic; amplitude: 0–10 mA; duration: 0.2 ms; frequency: 1 Hz) using a handheld bipolar hook electrode (Rochester Electro-Medical, Lutz, FL; #400900) 5 mm proximal to the repair zone. The stimulus intensity was increased to obtain a supramaximal CMAP and averaged over a train of 5 pulses with 1 s intervals between each pulse. CMAP recordings were amplified with 100× gain and recorded with 10–10,000 Hz band pass and 60 Hz notch filters.

Compound nerve action potential recordings were obtained across the graft by stimulating at the proximal site of the sciatic nerve and the distal site on the common peroneal nerve (biphasic; amplitude: 0–1 mA; duration: 0.2 ms; frequency: 1 Hz; 1000× gain, bandpass filter: 10–2000 Hz). The proximal site was stimulated with a handheld bipolar hook electrode (Rochester Electro-Medical, Lutz, FL; #400900) and recorded distal to the repair zone with a bipolar electrode (Medtronic, Jacksonville, FL; #8227410). The response was averaged over a train of 5 pulses with 1 s intervals between each pulse. The ground electrode (Medtronic, Jacksonville, FL; #8227103) was inserted into subcutaneous tissue halfway between the electrodes.

### Nerve Harvest and Histology

At time of harvest, rats were overdosed with sodium pentobarbital. The entire length of the repaired nerves was harvested and placed in 4% paraformaldehyde for 48 h at 4°C. The excised tissue was immersed in 30% sucrose solution for 48 h or until fully saturated. For acute (2 week) animals, the entire repair zone and distal nerve segments were cryosectioned longitudinally (20–25 μm thickness), mounted on glass slides, and immunolabeled using antibodies listed below. For chronic (12 or 16 week) animals, nerve cross-sections were stained and analyzed. Here, the nerve was blocked 5 mm distal to the repair zone and embedded in paraffin. Axial sections (thickness: 8 μm) were taken with a microtome, mounted on glass slides, deparaffinized in xylene and rehydrated with a descending gradient of ethanol. Following rehydration, antigen retrieval was performed in TRIS/EDTA buffer using a modified pressure cooker/microwave technique. Briefly, slides were placed in Citrasolv and put in an oven at 60°C for 20 min, sequentially rinsed in 100 and 95% ethanol, placed in TRIS/EDTA buffer within a microwave safe pressure cooker (Nordic Ware tender cooker product # 62104) and microwaved for 8 min, after which the slides were allowed to cool for 10 min and then rinsed in 1× PBS/TWEEN. Next, normal horse serum in Optimax (BioGenex) was applied per manufacturer’s instructions (VectaStain Universal Kit) and sections were incubated at 4°C with primary antibody (see list below) in Optimax and normal horse serum (VectaStain Universal kit). After washing the sections three times for 5 min with PBS/TWEEN, an appropriate fluorescent secondary antibody was applied for 1 h at room temperature. After rinsing three times, sections were cover slipped. Longitudinal and/or axial sections were labeled using the following primary antibodies (i) SMI31/32 (neurofilament, 1:1500 frozen, 1:1000 paraffin, Covance Research Products), (ii) SMI35 (neurofilament, 1:500, Covance Research Products), (iii) neurofilament-200kDa (1:200, Sigma), (iv) S-100 (1:250, Dako), and/or (v) myelin basic protein (frozen: SMI-94R, 1:500, Covance Research Products; paraffin: CPCA-MBP, 1:1500, Encor). The following secondary fluorophore-conjugated antibodies (Alexa Fluor – 488, 568, and 647; or Jackson ImmunoResearch) were used as appropriate.

For muscle mass measurements, immediately after euthanasia the ipsilateral and contralateral tibialis anterior muscles were carefully removed by cutting the distal tendons, and weighed to obtain the wet muscle mass. Percent recovery was calculated by normalizing the wet muscle mass to the contralateral side.

### Imaging, Quantification, and Statistical Analyses

#### Microscopy

The sections were examined under an epifluorescent microscope (Eclipse E600; Nikon, Melville, NY) and the images were digitally captured (Spot RT Color; Diagnostic Instruments, Sterling Heights, MI). Alternatively, the sections were fluorescently imaged using a laser scanning confocal microscope (AR1, Nikon or LSM 710, Zeiss).

#### Acute Regeneration

**FIGURE 2:**
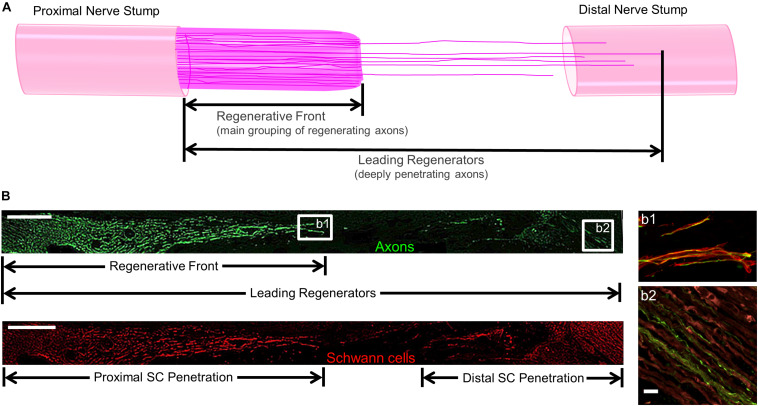
Methods to Quantify Axon Regeneration and Schwann Cell (SC) Infiltration. **(A)** Cartoon depicting the staggered process of acute axonal regeneration across a segmental nerve lesion, where the main bolus of regenerating axons – termed the “Regenerative Front” – is preceded by an accelerated, but less dense, population of regenerating axons – termed the “Leading Regenerators.” These 2 populations of regenerating axons were measured for each animal at 2 weeks following repair of 1 cm segmental defects by quantifying the distance from the proximal end of the repair zone to the distal axonal projections, with multiple sections surveyed per animal to ensure that the maximal values were attained. **(B)** Examples of longitudinal tissue section of nerve regeneration across a 1.0 cm sciatic nerve gap (repaired by a TENG), with the repair zone beginning proximally on the left and proceeding distally to the right. (**B**, top) Example of axon regeneration measurements: neurofilament labeled with SMI31, and the measurements of the “Regenerative Front” and “Leading Regenerator” axons are denoted. Scale bar: 1000 μm. (**B**, bottom) Example of SC infiltration measurements: SCs are labeled with S100, and the measurements of both the proximal and distal infiltration are shown. Scale bar: 1000 μm. **(b1)** “Regenerative Front” showing colocalization of axons and SCs. **(b2)** “Leading Regenerator” axons found beyond the repair zone, within the distal stump. **(b1,b2)** Scale bar: 20 μm.

For regenerative measurements, sections were captured using the tilescan function in Zen (Zeiss). Regeneration measurements were made by two experienced technicians who were blinded as to the repair group whenever possible, although histological features such as the presence/absence of NGT remnants and presence/absence of TENG neurons/axons made complete blinding impossible for all cases. Acute axon regeneration (at 2 weeks post-repair) was measured across multiple longitudinal sections (minimum of 3 levels) to quantify the distance of the “Regenerative Front” (RF; defined as the main bolus of regenerating axons, always still within the graft zone at 2 weeks following repair of a 1 cm lesion) and “Leading Regenerators” (LRs; defined as the furthest penetrating axons out ahead of the RF axons, generally in the distal stump following autograft or TENG repair, but within the graft zone following NGT repair) (Figure 2). RF and LR lengths were measured from the proximal coaptation site (denoted by the sutures in autograft repairs, and the proximal stump transition ∼1 mm in from the edge of the NGT following TENG or NGT repairs). Proximal and distal SC infiltration was also measured from the respective ends of the TENG/NGT to the most distal or proximal S100 staining originating from the respective stump (Figure 2). For images, multiple confocal z-stacks were digitally captured and analyzed. All confocal reconstructions were from full thickness z-stacks from sections 20–25 μm thick.

#### Chronic Morphometry

Axial sections taken distal to the repair zone were labeled for the axonal marker, neurofilament, and myelin (MBP), as described above, to count myelinated axons. The number of axons in each fascicle of each nerve was counted using Fiji by two experienced, blinded technicians. The total axon counts for each repaired nerve was calculated.

#### Functional Measurements

Compound muscle action potential peak-to-baseline amplitudes were measured and normalized to the contralateral side to calculate percent recovery for each animal. CNAP peak-to-peak amplitude were measured and normalized to the contralateral side to calculate the percent recovery for each animal. Following a normality test, the data was log-transformed as necessary to adjust for non-normality.

#### Statistical Analyses

For all quantitative measures, the group mean, standard deviation, and standard error of the mean were calculated for each group. All quantitative data was analyzed using a one-way ANOVA with repair group as the independent variable and outcome measure (e.g., axon regeneration, SC infiltration, CNAP amplitude, CMAP amplitude, and muscle mass) as the dependent variable. When significant differences were detected between groups, Tukey’s *post hoc* comparisons test was performed for most cases with the exception of the functional data where Dunnet’s multiple comparisons test was performed. For all statistical tests, *p* < 0.05 was required for significance. Statistical testing was performed using GraphPad Prism version 7.03 (GraphPad Software, La Jolla, CA, United States).

## Results

### TENG Biofabrication and Characterization

TENGs were generated within custom-built mechanobioreactors via the controlled separation of integrated neuron populations. DRG explants were isolated from embryonic rats, plated on a stationary membrane and a movable overlapping “towing” membrane, and then virally transduced to express green fluorescent protein (GFP) or mCherry to permit subsequent *in vivo* identification. The two neuronal populations formed connections via axonal extensions across the two membranes over the course of the first 5 days *in vitro* (Figure 1). Then, the towing membrane was slowly pulled away, driven by a precise computer-controlled stepper motor, to physically elongate the axon tracts in micron-scale increments. As previously described, the axon tracts responded to these forces by adding new axon constituents (e.g., cytoskeleton, axolemma, organelles, etc.), increasing diameter, forming fascicles, and lengthening to create long tracts of living axons (Figure 1; Pfister et al., 2004, 2006; Huang et al., 2009; Smith, 2009). In this manner, axons were stretch-grown to lengths of 1 or 2 cm in length over the next 7 or 14 days *in vitro*, respectively. It is important to note that the stretch-growth media includes specific mitotic inhibitors that have been shown to effectively remove the presence of glia; thus, TENGs are virtually exclusively comprised of DRG neurons and their long axon tracts. Three-dimensional TENGS were then created by encapsulating these stretch-grown living axonal tracts in a collagenous matrix and removing them as a whole for transplantation within a premeasured NGT (Pfister et al., 2006; Huang et al., 2009). For implantation, the rat sciatic nerve was exposed and a segment was excised and replaced with an autologous nerve graft (180° reversal of excised nerve), a TENG (within an NGT), or an NGT (either empty or filled with the collagenous matrix used to encapsulate TENGs).

### Acute TENG Mechanism of Action and Efficacy

**FIGURE 3:**
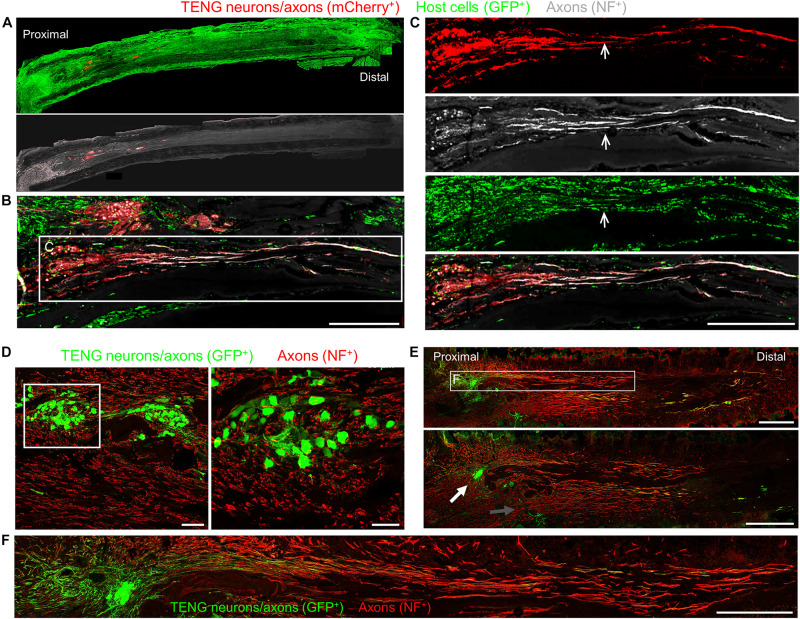
TENG Survival, Maintenance of Architecture, and Mechanism-of-Action following Allogeneic Transplants in Rats. Longitudinal sections across the graft zone at 2 weeks post-implant. **(A–C)** Implant of mCherry + TENGs into GFP + host rats to discriminate TENG neurons/axons (red) versus host cells/axons (green). **(A)** Full-width longitudinal section and **(B)** zoom-in of region of interest showing robust and directed host axon regeneration and support cell infiltration directly along TENG axon tracts. **(C)** Region-of-interest showing individual channels depicting TENG neurons/axons (red), axons (purple), all host cells (green), with overlay. Arrow depicts TENG axons co-localized with host axons, suggesting directed growth. Scale bars **(A)** 500 μm; **(B)** 250 μm; **(C)** 300 μm. **(D–F)** GFP + TENG neurons/axons into wild-type host rats. **(D)** Surviving TENG neurons/axons (green) exhibiting healthy morphology (scale bar: left, 100 μm; right 50 μm). **(E,F)** A nerve repaired using a TENG (GFP +) labeled with SMI31 (red) to show host axon regeneration. **(E)** Living TENG neurons and axons (green) were found across the entire nerve graft. TENG neurons/axons were placed off-center to demonstrate that host axons have a preference to follow the path created by the stretch grown axons: the white arrow points out the altered direction of host axon growth through the main cluster of TENG cell bodies and axons; the gray arrow points out the natural axon regeneration trajectory straight out from the proximal stump (scale bars: 1000 μm). This suggests that TENG neurons/axons actively direct and guide host axon regeneration. **(F)** Region-of-interest showing dense bundles of host axons that were intertwined with and appeared to grow directly along TENG axons. Collectively, these images provide evidence of a new mechanism of nerve regeneration: axon-facilitated axon regeneration (AFAR) denoted by host axon regeneration directly along tissue engineered axon tracts.

To assess the acute regenerative response that occurred in animals treated with TENGs versus autografts or NGTs, at 2 weeks after implantation the nerve/graft zones were harvested and immunohistochemical examination was performed on longitudinal frozen tissue sections. Based on fluorescent reporter expression (i.e., mCherry + TENG neurons/axons in GFP host rats or GFP + TENG neurons/axons in wild-type host rats), microscopic examination of tissue sections revealed histological evidence of surviving transplanted DRGs and maintenance of the aligned axonal architecture within all TENG transplants, as expected (Figures 3A–C). To assess host axon regeneration into the graft zones, sections were immunolabeled for SMI31/32, labeling neurofilament isoforms broadly expressed by regenerating host axons but not generally expressed by TENG DRG neurons/axons likely due to maturation state of these axons during and immediately following the stretch-growth process. These longitudinal sections provided evidence that TENGs were actively facilitating the axon regeneration process, rather than simply behaving as a permissive substrate for regeneration. In particular, when TENG neurons and axons were placed off-center, host axons altered their growth direction (Figure 3E, white arrow) instead of strictly following the proximal stump trajectory (Figure 3E, gray arrow). This suggests that TENG neurons and axons actively directed and guided the host axon regeneration. Additionally, we employed high-resolution confocal microscopy to enable direct visualization of the mechanisms of axon regeneration at the micro-scale (i.e., visualizing cell-axon and axon-axon interactions). We found regenerating axons appeared to have an intrinsic preference to be guided by TENG axons, thus facilitating accelerated host axon regeneration (Figures 3D–F). We refer to this mechanism as “axon-facilitated axon regeneration” (AFAR), denoting a potentially intrinsic ability of TENG axons to promote host axon growth directly along their structure. It is important to note that while this method of structural imaging suggests that host axons follow a path along TENG axons, this strategy does not indicate the underlying cell-cell receptors and/or secreted factors facilitating this mechanism of axonal re-growth.

**FIGURE 4:**
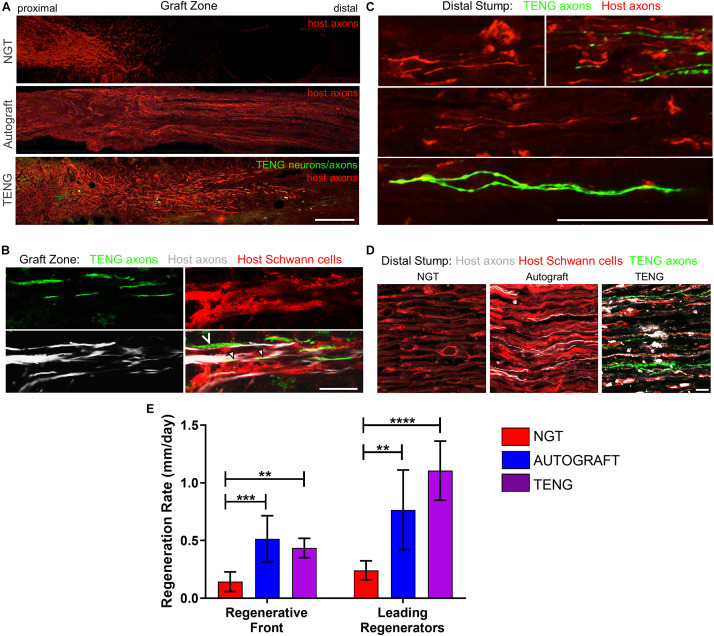
TENGs accelerate host axon regeneration. Longitudinal sections **(A,B)** across the graft zone and/or **(C,D)** into the distal stump at 2 weeks following repair using an NGT, a reverse autograft, or TENG. **(A)** Axon regeneration as labeled by SMI31 (red, host axons) and GFP (implanted TENG neurons/axons) showing the “regenerative front” – main bolus of regenerating axons – for each repair group. Regeneration through an NGT yielded retarded and disorganized axonal extension. Regeneration through a reverse autograft was highly organized owing to the presence of aligned autologous SCs throughout the graft (not shown). Regeneration through TENGs showed host axons following the length of the GFP-labeled TENG axons, with greater penetration and more organization than that found following NGT repair. Scale bar: 1000 μm. **(B)** Clear discrimination of TENG and host axons, as well as host SCs, across the graft zone. In addition to direct AFAR (hollow arrowhead), host SCs also migrated and organized along TENG axons (solid arrowheads), creating a “tripartite” regenerative complex of TENG axons: host axons: host SCs. Scale bar: 10 μm. **(C)** Following TENG repair, a group of host “Leading Regenerator” axons was present in the distal stump – far afield from the “Regenerative Front” – and were seen along with TENG axons that had also penetrated into the distal stump. Scale bar: 50 μm. **(D)** These accelerated “Leading Regenerator” axons were present following TENG or autograft repair, but were always absent following NGT repair. **(E)** Quantification of axon regeneration (mean ± standard deviation). Host “Regenerative Front” and “Leading Regenerators” were statistically equivalent following TENGs and autografts repair, and both were superior to NGT repair (**p < 0.01, ***p < 0.001, ****p < 0.0001).

The extent and rates of axon regeneration across TENGs, NGTs, and autografts were also measured. Host axon penetration across TENGs was greatly increased in comparison to NGTs and was similar to that attained by autografts (Figures 4A,D,E). In our evaluation of the acute axon regeneration process, we observed two distinct axonal factions: a major bolus of regenerating axons which we termed the “Regenerative Front” (RF), and a much smaller group of more rapidly regenerating axons out ahead of the regenerative front, which we termed “Leading Regenerator” (LR) axons (Figures 4A,C). Notably, the discovery of LR host axons in the distal stump was only found following TENG or autograft repairs, not following NGT repairs (Figures 4C,D). These distinct LR and RF populations of regenerating axons were quantified for all animals at 2 weeks following repair of 1 cm lesions (see Figure 2 for a description of the methods). This analysis determined that TENGs and autografts yielded an equivalent rate of host axon regeneration across the lesion, and that this rate was superior to that observed in animals treated with NGTs (Figure 4E). Specifically, TENGs were statistically equivalent to autografts for the RF (*p* = 0.59), with a trend toward TENGs enhancing host LR axon penetration (*p* = 0.081). Based upon the lengths of both the RF and the LRs, TENGs significantly accelerated axonal regeneration versus NGTs (both *p* < 0.01), resulting in axon growth rates 3- to 4-fold faster (peaking at >1 mm/day across the graft zone) than NGTs (Figure 4E). We also assessed the density and directionality of axonal regeneration at various locations along the repaired nerve and/or graft. As expected, similar axonal density and morphology was observed across groups in the proximal nerve segment. At the regenerative front, autografts exhibited the greatest longitudinal directionality, while axonal regeneration across TENGs appeared slightly less organized, and NGTs displayed a disorganized webbed morphology, especially at the leading front (Figure 4A). Also, NGT axonal density dropped off sharply at the RF, but was more tapered for TENGs and autografts. Interestingly, along with evidence of host axons guided directly along TENG axons, host Schwann cells were also seen to migrate onto the axons, creating a regenerative complex consisting of TENG axons, host Schwann cells, and host axons (Figure 4B).

Distal to the graft zone, SCs forming the bands of Büngner were found in all groups (Figure 4D). However, in the NGT group, there were no axons found in the distal nerve segment, indicating that no axons had crossed the NGTs at 2 weeks post-repair. In contrast, numerous axons were found in the distal nerve structure in animals treated with autografts and TENGs at this early time point (Figures 4C,D). These deeply penetrating host axons within the distal nerve resembled each other in density and morphology for autograft and TENG repairs. Notably, in the TENG group, TENG axons were also found in the distal nerve along with host axons (only host axons were measured as LRs). There were roughly twice as many TENG axons (GFP +) compared to host axons (GFP-) in the distal nerve, demonstrating the permissive environment for axon outgrowth (Figures 4C,D).

**FIGURE 5:**
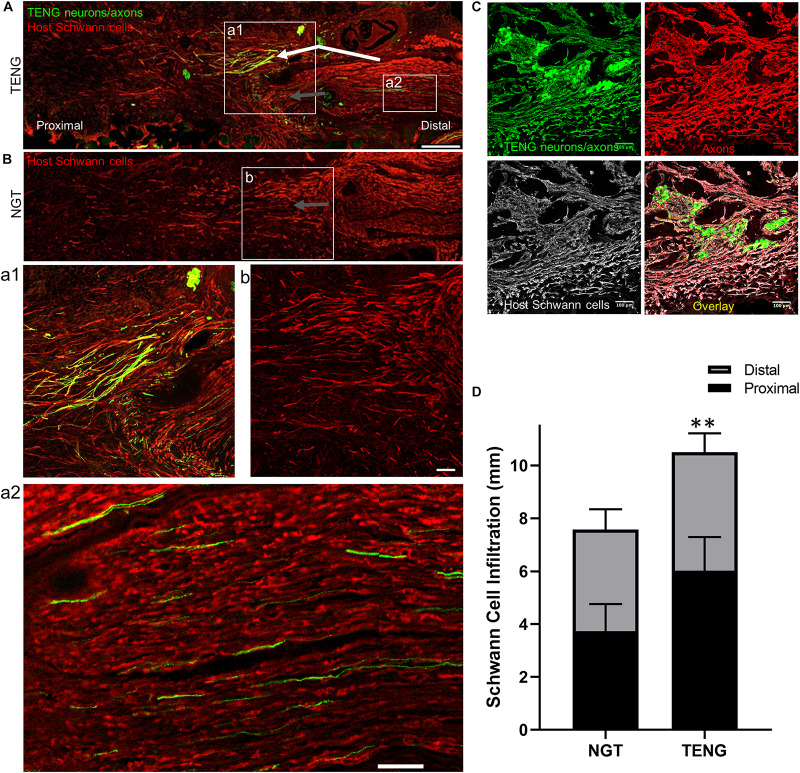
TENGs Direct Host Schwann Cell Infiltration. Longitudinal sections across the graft zone at 2 weeks following repair using TENGs or NGTs. **(Aa1)** In cases where TENG axons (green) were present off-center (shown in upper half of the boxed section), SC infiltration was markedly directed upward toward TENG axons, indicating that SCs are attracted to TENG axons and alter their migration to infiltrate along TENG axons. **(Bb)** In NGTs, SCs generally infiltrated linearly from ends, typically observed only in center of NGT (tapered cone). **(a2)** TENG axons also projected into the distal nerve stump to grow along host SCs. **(A,B)** Scale bars: 500 μm. **(a1,a2,b)** Scale bars: 100 μm. **(C)** Host SCs also directly interacted with TENG neurons; scale bar: 100 μm. **(D)** Plot of quantified SC infiltration measurements (mean ± standard deviation). SC penetration from the proximal and distal ends was quantified. TENGs significantly increased infiltration of SCs compared to NGTs (**p < 0.01).

As SCs are traditionally believed to be the major drivers of host axonal regeneration across nerve lesions, we also examined the effects of TENGs on host SC infiltration and organization to further understand the effects TENGs had on the active nerve regeneration process. We measured the infiltration/migration distance of SCs from the host tissue, both proximally and distally, into the TENGs and NGTs; such measurements were not warranted for autografts, since they have a homogenous distribution of host SCs from the onset. We found that SC infiltration distance was enhanced in animals treated with TENGs (Figure 5). Conversely, animals that received NGTs showed modest SC penetration from both the proximal and distal ends, with a clear SC-free gap near the center in all cases (Figure 5B). Indeed, reconnection of proximal and distal SCs was observed in TENGs but not in NGT groups. In TENGs, the SCs appeared in structures resembling “cables,” potentially precursors to the formation of intra-graft bands of Büngner (Figure 5A). Overall, TENGs enhanced SC infiltration into the nerve gap versus NGTs (*p* < 0.01) (Figure 5D).

Similar to the findings with axon directionality, we observed that SCs had a strong preference to migrate directly to TENG neurons and then directly along TENG axonal tracts (Figures 5A,C). This was likely driven by cell-cell guidance mechanisms, and demonstrated that TENGs actively influenced, directed, and most importanty, accelerated SC infiltration. Taken together, these findings suggest that TENGs play an active role in accelerating the natural regeneration process by encouraging SC infiltration and alignment.

### Chronic Functional Recovery and Nerve Morphometry

**FIGURE 6:**
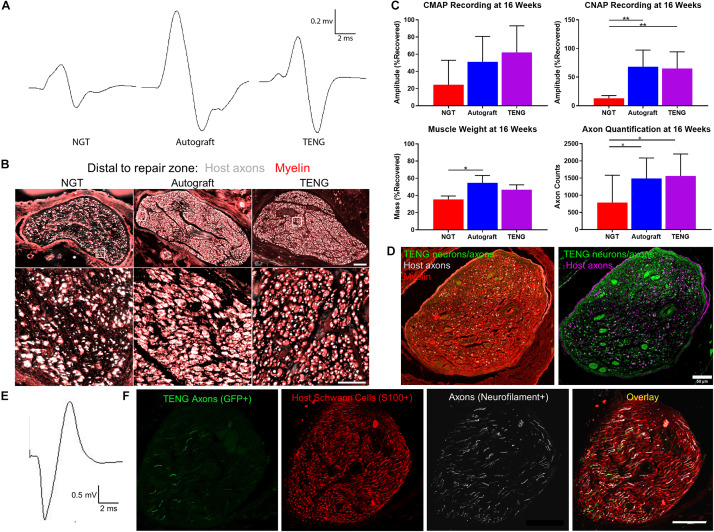
TENGs Facilitate Functional Recovery. **(A–C)** Functional recovery and structural regeneration at 16 weeks following repair of 1 cm nerve lesions using NGTs, reverse autografts, or TENGs. **(A)** Representative CMAP traces. **(B)** Representative nerve morphometry showing nerve cross sections (5 mm distal to repair zone) labeled for axons (red) and myelin (purple). Scale bar (top): 100 μm; scale bar (bottom): 50 μm. **(C)** Plots of mean recovery levels for CMAP, CNAP, muscle weight, and axon density (mean ± standard deviation; *p < 0.05 and **p < 0.01 versus NGT). **(D–F)** Proof-of-concept data showing structural and functional regeneration at 12 weeks following repair of 2 cm nerve lesions using TENGs. **(D)** Chronic nerve morphometry showing representative nerve cross sections (5 mm distal to repair zone) labeled for axons (purple) and myelin (red), also showing TENG neurons and axons (green). Scale bar: 50 μm. **(E)** Example CNAP across a 2 cm nerve segment repaired using a TENG. **(F)** Oblique nerve cross section (5 mm distal to repair zone) showing the architecture of numerous TENG axons (GFP+, green) interacting with host SCs (S100+, red) and growing along other axons (NF+, purple). Scale bar: 100 μm. **(A,E)** Electrophysiological traces were averaged over a train of 5 pulses with 1 s intervals between each pulse, with band pass filtering from 10 to 10,000 Hz for CMAP and 10–2000 Hz for CNAP.

We also assessed the degree of functional recovery and mature axonal regeneration at 16 weeks following repair of 1 cm nerve lesions using NGTs, reverse autografts, or TENGs. We found evidence of muscle reinnervation in all animals at this time point based on the presence of compound muscle action potentials (CMAPs); however, the shape and amplitude of the CMAP traces indicated improved muscle health and reinnervation following TENG or autograft repair versus NGT repairs (Figure 6A). The extent of nerve and muscle recovery as measured by CMAP amplitude (normalized to the contralateral muscle) demonstrated a trend toward improvement following TENG or autograft repair versus NGT, but there were not statistically significant differences. Similarly, there was a trend toward improved muscle mass following TENG repair versus NGT, but muscle mass was only significant for autograft versus NGT repairs (*p* < 0.01). In contrast, compound nerve action potential (CNAP) measurements demonstrated a more robust nerve functional recovery following TENG or autograft repairs versus NGT repairs (Figure 6C; each ^∗∗^*p* < 0.01). Nerve morphometry also revealed an increase in the density of regenerated host axons for TENG and autograft repair versus NGTs (Figure 6B; each ^∗^*p* < 0.05). Overall, both TENG and autograft repairs elicited superior levels of functional recovery and axon regeneration across multiple metrics as compared to that attained by NGTs, signaling the benefits of endogenous or engineered living scaffolds.

Finally, in a proof-of-concept study, we also assessed the ability of TENGs to facilitate axonal regeneration and functional recovery when used to bridge challenging 2 cm defects. Demonstrating proof-of-concept in a longer lesion in rats is a crucial challenge for a pre-clinical repair strategy. While most engineered bridging solutions are successful for 1 cm lesions due to the strong regenerative capacity of young adult rats, 2 cm defect sizes are generally considered to be beyond the critical defect length in rats and therefore only a subset of effective treatments at shorter (1 cm) defects are effective at longer (2 cm) defects. As we did not know the time course of regeneration and reinnervation for TENGs when used to bridge 2 cm defects, we assessed functional recovery over time using evoked muscle/foot twitch, CMAP, and/or CNAP measurements. This revealed that sciatic nerve stimulation evoked muscle function in some animals as early as 12 weeks following repair with TENGs, with a higher proportion at 16 weeks post-repair. Nerve morphometry analyses supported these functional measures, revealing dense axonal regeneration following TENG repair (Figures 6D,F). Consistent CNAPs across the repair zone were also apparent by 12 weeks (Figure 6E). Moreover, TENG axons were also found in the distal nerve out to at least 3–4 months post-repair. These promising results suggest that TENGs may scale to longer lesions and provide regenerative benefits.

## Discussion

Peripheral nerves have a limited capacity for regeneration following injury; however, there is a significant need for next-generation tissue engineered constructs to augment endogenous regenerative mechanisms across segmental nerve defects. To address this need, we have developed living TENGs that consist of aligned axonal tracts routinely generated through the controlled process of axon “stretch-growth.” The concept of TENGs is inspired by developmental neurobiology, where targeted axon outgrowth occurs directly along pathways blazed by existing axon tracts, termed “pioneer” axons. When used to bridge segmental sciatic nerve defects in rats, we found that the degree of axonal regeneration and functional recovery attained following TENG repair was statistically equivalent to the “gold standard” autograft repair and consistently superior to NGTs. The proposed MoA of TENGs, which facilitates accelerated axon regeneration across the graft via AFAR as well as accelerated SC infiltration, is the key differentiator from existing acellular bridging strategies. Indeed, TENGs proved to be superior to NGTs by all three acute metrics measured: host RF axonal regeneration, presence of LR axons in the distal stump, and degree of SC infiltration. In turn, TENGs resulted in several fold increases in the extent of functional recovery following TENG repair versus NGT repair.

The technique of autologous nerve grafting is considered the “gold standard” and the most reliable choice in repair of major defects in peripheral nerves (Chiu and Ishii, 1986; Kline and Hudson, 1995; Lundborg, 2000). The introduction of an autologous nerve segment provides physical and biological scaffolding to guide regenerating axons extending to appropriate targets within the periphery. However, complications with the use of autografts exist and are due to the limited supply of donor nerves and the likelihood of donor site morbidity and vulnerability to infection (Chiu and Ishii, 1986; Kline and Hudson, 1995; Lundborg, 2000). To address the limitations of autografts, alternative strategies to repair damaged nerves have implemented materials of biologic or synthetic origin (Hudson et al., 2000; Scherman et al., 2001; Cheng and Chen, 2002; Meek and Coert, 2002). Currently in clinical use are biomaterial-based tubes, such as those comprised of PGA or collagen. These conduits act as a physical guide for SCs migrating from the proximal and distal nerve stumps, and in turn for axons sprouting from the proximal nerve stump to reach the disconnected nerve segment, which then provides chemical and physical cues to direct regenerating axons to ultimately reinnervate the target tissue (Lee and Wolfe, 2000; Hall, 2001; Belkas et al., 2004). However, synthetic conduits have only been clinically successful for the repair of short nerve lesions, and they are typically used for gaps less than 1 cm close to the end target (Evans, 2000; Meek and Coert, 2002). Nerve allografts have shown promise in facilitating regeneration across nerve defects by acting as a tissue scaffold while the native nerve fibers regenerate. However, living allografts require immunosuppression in order to evade a detrimental host immune response. To combat this, acellular nerve allografts (ANAs) have been developed which do not require immune suppression and work largely by promoting host Schwann cell infiltration (Palispis and Gupta, 2019). Regardless of whether a segmental defect is grafted with a donor nerve or a synthetic conduit, the axons and many supportive cells of the disconnected portion of the nerve ultimately degenerate (Lee and Wolfe, 2000; Hall, 2001; Belkas et al., 2004). Due to the relatively slow growth of sprouting axons – as slow as 0.1–0.2 mm/day across an NGT and approximately 1 mm/day across an autograft – as well as the gradual loss of the distal pathway necessary to guide axon outgrowth in the distal segment (Lee and Wolfe, 2000; Hall, 2001; Belkas et al., 2004), poor functional recovery of extremities that are far away from nerve damage is often seen regardless of the clinically available repair strategy used (Evans, 2000; Meek and Coert, 2002). An example of this is commonly found with brachial nerve injury, where elbow flexion may ultimately be regained, but hand function generally is not (Gutowski and Orenstein, 2000).

Over the past few decades, alternative tissue engineered solutions have been sought to overcome the limitations associated with autografts and NGTs. These approaches include creating combinations of permissive scaffolds (such as decellularized grafts or hydrogels), extracellular matrix (ECM), trophic factors, and glial or stem cells (Höke et al., 2003). Several groups are investigating the use of growth factors and/or Schwann and glial cell combinations to enhance nerve growth within nerve guidance channels (Evans et al., 2002; Frerichs et al., 2002; Lee et al., 2003; Yu and Bellamkonda, 2003; Fansa and Keilhoff, 2004; Hu et al., 2005; Stang et al., 2005; Chalfoun et al., 2006; Keilhoff et al., 2006; Nie et al., 2007). Yet others have shown that axons appear to prefer longitudinally aligned fibers (Bellamkonda, 2006; Kim et al., 2008) as a regenerative substrate, and attempts are being made to create fibers that elute trophic factors (Cao et al., 2009). Unfortunately, these approaches typically ignore the fact that axonal outgrowth *in vivo* occurs along SCs and the basal lamina; thus, strategies that were optimized based on directly promoting axonal outgrowth *in vitro* generally do not mechanistically translate *in vivo*. In addition, these approaches all focus on promoting regeneration of the proximal stump; none of these strategies can delay the eventual degeneration of the distal pathway. Our transplantable, scalable nervous tissue constructs generated via axon “stretch-growth” represent a promising strategy to augment and accelerate endogenous regenerative mechanisms following nerve damage. The TENG strategy is an attempt to couple the benefits of the above mentioned approaches by using tissue engineered axon-based living scaffolds that can potentially provide structural and trophic support not only to serve as a bridge for regenerating axons, but also extend axons into the otherwise axotomized distal nerve segment to potentially maintain the pro-regenerative environment.

Our findings suggest that TENG axons – via AFAR – mimic the action of “pioneer” axons during development and thereby may provide a combination of physical and neurotrophic support to regenerating host axons. Indeed, living axons have been shown to play a critical role during embryonic development in ensuring proper axonal targeting and driving widespread connectivity, and thus may be an essential element of any tissue engineering approach designed to exploit developmental mechanisms in the context of axon regeneration and targeting. While the techniques of differential labeling (e.g., GFP TENGs in wild-type rats and mCherry TENGs in GFP rats), immunohistochemistry, and confocal imaging used in the current study provided compelling data showing co-localized growth of host axons along TENG axons, such visual evidence alone does not demonstrate the actual underlying molecular mechanism(s). Indeed, the molecular mediators of AFAR are likely a combination of axon-axon linkages and locally secreted growth factors, and will require the execution of non-trivial receptor blocking and/or soluble factor attenuation studies which will be the subject of future investigations. In addition, we exploited the observation that TENG axons do not express SMI31/32 to aid in differentiating host versus TENG axons. While TENG axons are reactive to other neuron-specific cytoskeletal proteins, we believe the lack of SMI31/32 immunoreactivity may be due to the maturation of the TENG axons throughout and immediately after the “stretch-growth” process. We are conducting ongoing studies to investigate cytoskeletal maturation of stretch grown neurons/axons to ascertain at what time point after stretch-growth has completed that these axons begin to express SMI31/32. Moreover, it is important to note that the presence of SCs pre-added to TENGs would likely be detrimental to the mechanism of AFAR, since pioneer axons in development are not myelinated, and the addition of SCs would likely make TENGs highly immunogenic. However, if TENGs were made using autologous cells it would be useful to compare the efficacy of SC-seeded TENGs versus our standard SC-devoid TENGs.

This study demonstrated that our 3-D living TENGs actively facilitate axon regeneration by serving as a guide for host regenerating axons, referred to as AFAR. Through *in vivo* transplantation studies, we observed that host axons appeared to be growing directly along transplanted TENG axons, resulting in increased rates of regeneration compared to groups treated with NGTs, and statistically equivalent rates of regeneration compared to autograft repair groups. Most notably, a small group of axons that have infiltrated furthest into the graft zone or distal nerve stump, referred to as leading regenerators, were observed following TENG or autograft repair. This unique population of deeply penetrating axons – from both host and TENGs – may extend the living labeled pathway via a combination of neurotrophic support and contact guidance and therefore further promote regeneration. Indeed, these axons may serve to condition the distal environment for the arrival of follower? axons. Similar to the natural developmental process of the PNS, these axons may prescribe the initial path for subsequent regenerating axons to follow in a process known as “selective fasciculation”(Tessier-Lavigne and Goodman, 1996; Yu and Bargmann, 2001; Dickson, 2002). Moreover, these axons may serve as regenerative scouts, navigating the more distal environment and sending signals back to the regenerative front in preparation for the territory to come. However, further studies are necessary to test these potential attributes of exogenous axons projecting from implanted TENG neurons.

In addition to increased rates of axon regeneration, robust Schwann cell infiltration with formation of Bands of Bungner was observed, likely encouraging host axons to penetrate deep into distal nerve structures. Of note, we found that Schwann cells preferentially migrated to transplanted TENGs and appeared to migrate directly along TENG axons. This is a critical finding given that following the transection of a nerve, there is a small timeframe during which reinnervation needs to occur for recovery to be complete (Gutmann and Young, 1944; Fu and Gordon, 1995). During this same time, distal axons – which have been physically cut off from their neuronal cell bodies – undergo a gradual degenerative process and begin to experience a reduction of neurotrophic support leading to aggressive macrophagic degradation (Petrov et al., 2016). By promoting SC infiltration and organization into aligned columns, TENGs are likely able to promote the synthesis of neurotrophic factors required for regeneration, such as NGF, BDNF and NT-3 (Heumann et al., 1987; Thoenen et al., 1988; Funakoshi et al., 1993; Fu and Gordon, 1995; Raivich and Makwana, 2007; Petrov et al., 2016), which would accelerate the reformation of the SC basal lamina (Sunderland, 1968). This neurotrophic and structural support would in turn help to encourage axonal sprouts and likely expand the critical timeframe for reinnervation, thereby increasing the likelihood of functional recovery post-repair.

Increased axon regeneration and robust Schwann cell infiltration into and across the graft zone complement our functional recovery findings measured via CNAP and CMAP at both non-critical and critical nerve defect lengths. Overall, following repair of non-critical lesion (i.e., 1 cm), TENGs were seen to outperform NGTs, but remained statistically equivalent to autografts, in promoting functional nerve regeneration. This was not surprising considering the historical performance of the autograft for small lesions and the fact that the reverse autograft used in this study is a perfect geometric and modality (i.e., ratio of sensory to motor axons) match of the repaired nerve, which is not the case for a human autograft where a sensory nerve is generally used to repair a motor nerve and/or a size mismatch may occur due to limited autograft availability. While the proof-of-concept studies evaluating the performance of TENGs following repair of a critical nerve gap (i.e., 2 cm) revealed successful host axonal regeneration, nerve conduction, and reinnervation, this study lacked comparison to the autograft. However, these promising results using TENGs to bridge 2 cm lesions provide a foundation for TENG scale-up and additional testing in long-gap nerve injury models in comparison to clinical standards.

**FIGURE 7:**
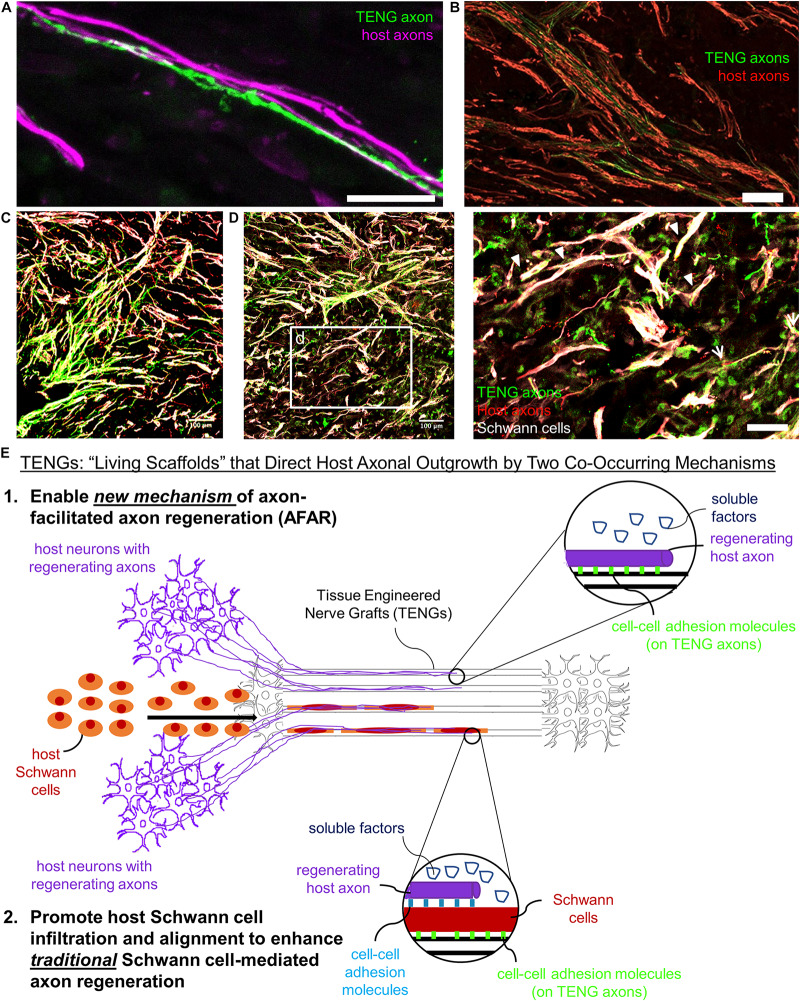
TENGs Mechanisms-of-Action: The Value of Axons. Host axons favor paths created by transplanted TENG axons. TENGs serve as a living scaffold to facilitate regeneration via AFAR on the level of both **(A)** individual axons (scale bar: 100 μm) and **(B)** groups of axons (scale bar: 50 μm). **(C,D)** Tripartite regenerative mechanism: integration of TENG axons with both host axons and host SCs (scale bar: 100 μm). **(d)** Inset image from **(D)** showing colocalization of TENG axons with host axons and host SCs. TENGs enhance SC alignment as pointed out by the arrowheads, which then facilitate host axon growth. The arrows show instances of direct AFAR, illustrated by colocalization of TENG and host axons without the presence of host SCs (scale bar: 50 μm). **(E)** Conceptual schematic depicting the MoA of TENGs, leading to synergistic presentation of neurotrophic, chemotaxic and haptotaxic cues only possible with a “living scaffold.” Overall, TENGS possess novel mechanisms compared to NGTs & autografts: direct AFAR (not possible with autograft), increased SC infiltration and alignment (versus NGT), and robust presence of “leading regenerator” axons (versus NGT). This AFAR – unique to TENGs – compliments traditional SC-mediated axon regeneration.

Based on the current results, TENGs appear to possess a novel MoA compared to NGTs and autografts. TENGs serve as a living scaffold to facilitate nerve regeneration via two complimentary mechanisms (Figure 7): (1) *axon-facilitated axonal regeneration or AFAR:* TENGs accelerated host axon regeneration directly along TENG axons even in the absense of SCs, and (2) *enhancement of traditional SC-mediated axonal regeneration:* TENG axons increased host SC infiltration and alignment, which in turn accelerated host axon regeneration along these SCs. This newfound form of axon regeneration – AFAR – is unique to TENGs and complements traditional SC-mediated axon regeneration. The MoA of TENGs, which would intrinsically lead to synergistic presentation of neurotrophic, chemotaxic and haptotaxic cues, is only possible with a living scaffold. Notably, no alternative nerve repair approach (NGTs, autografts, acellular allografts) provides living axons to take advantage of the natural AFAR mechanism of regeneration.

Overall, we found that AFAR accelerates, directs, and enables robust host axon regeneration and appears to act in a complimentary manner to traditional SC-mediated axon regeneration. Of note, the mechanism of AFAR does not apply to autografts since the host axons initially within an autograft will inevitably degenerate due to the action of excising the autograft from its original site. This suggests that TENGs may have the potential to attain robust axon regeneration without a dependence on proliferating SCs from host tissue, possibly negating the suggestion that SC senescence may inherently lead to a limited axon regeneration across major (>4 cm) nerve injuries (Saheb-Al-Zamani et al., 2013). Additionally, the outgrowth of TENG axons into the distal nerve stump may enable TENGs to maintain distal nerve segment SCs in a pro-regenerative phenotype and therefore maintain efficacy of the distal pathway to provide a guide for regenerating host axons to reach long-distance targets; however, follow-on studies will be required to test this potential benefit of axonal ingrowth from TENGs.

## Author’s Note

This manuscript has been released as a pre-print in bioRxiv manuscript ID: 654723 (Katiyar et al., 2019).

## Data Availability Statement

The datasets generated for this study are available on request to the corresponding author.

## Ethics Statement

The animal study was reviewed and approved by Institutional Animal Care and Use Committees (IACUC) at the University of Pennsylvania and the Michael J. Crescenz Veterans Affairs Medical Center.

## Author Contributions

DC, ZA, HL, and DS conceived of and designed the experiments. KK and LS carried out TENG biofabrication and *in vitro* imaging/assessment. BC and JK designed and fabricated nerve guidance conduits. JB, JM, and KB performed lesion and implantation surgeries. JB, LS, JM, and KK performed electrophysiology assessments. JM, JB, and FL performed histological evaluations and confocal microscopy. KK, LS, JB, and KB analyzed the data. DC oversaw all studies and wrote the manuscript. All other authors provided edits and comments to the manuscript.

## Conflict of Interest

DC, DS, and HL are co-founders and KK is currently an employee of Axonova Medical LLC, which is a University of Pennsylvania spin-out company focused on translation of advanced regenerative therapies to treat nervous system disorders. Multiple patents relate to the composition, methods, and use of tissue engineered nerve grafts, including U.S. Patent 6,264,944 (DS), U.S. Patent 6,365,153 (DS), U.S. Patent 9,895,399 (DS and DC), and U.S. Provisional Patent 62/569,255 (DC). The remaining authors declare that the research was conducted in the absence of any commercial or financial relationships that could be construed as a potential conflict of interest.
